# Cost-to-baby as a percentage of median household income predicts population-level access to ART: a global health economics analysis

**DOI:** 10.1093/hropen/hoag084

**Published:** 2026-09-22

**Authors:** Giuseppe Silvestri, Steve Rooks, Alejandro Chavez-Badiola, Silvana Perez, Stephanie Kuku, Alan Murray, David Sable, G David Adamson

**Affiliations:** Conceivable Life Sciences, New York, NY, USA; Ontario Teachers Pension Plan, Toronto, ON, Canada; Conceivable Life Sciences, New York, NY, USA; Conceivable Life Sciences, New York, NY, USA; Conceivable Life Sciences, New York, NY, USA; Conceivable Life Sciences, New York, NY, USA; Special Situations Life Sciences and Life Sciences Innovation Fund, New York, NY, USA; Equal3 APC, Denver, CO, USA; Department of Obstetrics & Gynecology, Stanford University School of Medicine, Stanford, CA, USA

**Keywords:** assisted reproduction, epidemiology, infertility, reproductive decision making, affordability, utilization, fertility policy, socioeconomic determinants of health

## Abstract

**STUDY QUESTION:**

Does cost-to-baby, expressed as a percentage of median household income (HHI), predict population-level utilization of ART across countries?

**SUMMARY ANSWER:**

ART affordability follows a power-law relationship with population-level utilization (*R*^2^ = 0.77–0.84), with highest-utilization countries consistently achieving gross cost-to-baby below 100% and net out-of-pocket cost-to-baby below 50% of median HHI.

**WHAT IS KNOWN ALREADY:**

Cost has been consistently identified as a dominant barrier to ART access worldwide, and earlier cross-national work has shown that per-cycle IVF cost as a proportion of disposable income is associated with national utilization. However, no prior analysis has used the cumulative cost of achieving a live birth, normalized it to median HHI, tested a non-linear functional form, or separated gross from net (out-of-pocket) cost as independent predictors.

**STUDY DESIGN, SIZE, DURATION:**

Cross-national study using secondary aggregate data from ART registries and economic/demographic agencies for reporting years 2021–2023, covering 22 countries and regions that together represent more than 95% of estimated global ART cycle volume.

**PARTICIPANTS/MATERIALS, SETTING, METHODS:**

ART cycle volumes and live births were extracted from ICMART, ESHRE EIM, CDC NASS, CARTR, HFEA, REDLARA, ANZARD, and national registries. Median after-tax disposable HHI data were sourced from the Organisation for Economic Cooperation and Development (OECD) and World Bank, while female population aged 15–49 years and total live births were obtained from the United Nations Population Division. Gross cost-to-baby was calculated as the product of mean per-cycle cost (including embryo transfer, preimplantation genetic testing weighted by national usage, and medications) and the age-weighted number of cycles required to achieve a live birth. Net out-of-pocket cost-to-baby was derived after applying published reimbursement schedules, subsidies, and tax benefits, with structured estimates where authoritative published data were unavailable. Both metrics were expressed as a percentage of median HHI. Utilization was measured as the percentage of total national live births achieved via ART and as ART births per 1000 women aged 15–49 years. Power-law (log-log) regression was fitted by ordinary least squares; a subgroup analysis was performed for women aged 38–49 years. A Monte Carlo probabilistic sensitivity analysis (10 000 iterations; Program Evaluation and Review Technique (PERT) distributions with uncertainty bands of ±5%, ±7.5%, or ±10% calibrated to data-source quality) generated 90% simulation envelopes around each baseline regression.

**MAIN RESULTS AND THE ROLE OF CHANCE:**

Gross cost-to-baby varied more than 12-fold across countries, ranging from 66% of median HHI in Israel to 833% in Africa (excluding Egypt, Tunisia, and South Africa); net out-of-pocket cost-to-baby ranged from 13% to 825% of median HHI. Power-law regression demonstrated strong inverse relationships between affordability and utilization across all four model specifications (gross cost vs ART births per 1000 women aged 15–49 years: *R*^2^ = 0.84; net cost vs the same measure: *R*^2^ = 0.84; gross cost vs percentage of births via ART: *R*^2^ = 0.83; net cost vs percentage of births via ART: *R*^2^ = 0.77). The model-implied elasticity indicated that halving net out-of-pocket cost-to-baby is associated with a 2.67-fold increase in ART births per 1000 women of reproductive age and halving gross cost-to-baby with a 5.12-fold increase. Countries with gross cost-to-baby below 100% and net cost-to-baby below 50% of median HHI consistently achieved the highest utilization (e.g. Israel 8.7% of births via ART; South Korea 11.8%; Spain 11.7%; Japan 9.3%). The power-law relationship was preserved in women aged 38–49 years (*R*^2^ = 0.76–0.85). Monte Carlo simulation envelopes were narrow across all four models, indicating that the affordability–utilization gradient is robust to plausible variation in country-level input estimates.

**LIMITATIONS, REASONS FOR CAUTION:**

This is a cross-national, population-level (ecological) analysis. Since it uses aggregated, country-level data, the observed associations cannot support individual-level causal inference, may be subject to ecological confounding, and reverse causation through volume-driven economies of scale cannot be excluded. Registry data are heterogeneous across countries in reporting standards, cycle definitions, and completeness. Unmeasured confounders include cultural and religious attitudes, regulatory restrictions, clinic capacity constraints, and cross-border reproductive care. Net out-of-pocket cost estimates for a subset of countries were derived through structured triangulation that included AI-assisted synthesis and should be interpreted accordingly; the Monte Carlo analysis quantifies uncertainty in the affordability metrics but holds utilization outcomes fixed. The macro-level framework cannot address within-country inequities in ART access.

**WIDER IMPLICATIONS OF THE FINDINGS:**

Cost-to-baby relative to median HHI offers a standardized, policy-relevant affordability metric that builds on previous per-cycle approaches. The power-law relationship implies that affordability improvements yield the greatest marginal returns in countries where costs are highest relative to income. The independent predictive value of gross and net cost-to-baby identifies two complementary policy levers: provider-side efficiency (workforce expansion, technology adoption, and care-delivery efficiencies to bring gross cost-to-baby below 100% of median HHI) and demand-side coverage (insurance mandates, subsidies, or tax incentives to bring net out-of-pocket cost below 50%). Published generational accounting analyses indicate that the lifetime fiscal return on public ART investment exceeds its cost by 2.7- to 16-fold across 11 of 12 countries studied, supporting the underlying economic case.

**FUNDING:**

No specific funding was received; the study was supported by Conceivable Life Sciences.

**DISCLOSURES:**

G.S. is an employee and stock option holder at Conceivable Life Sciences. S.R. is an employee of Ontario Teachers’ Pension Plan, has received consultancy fees from Conceivable Life Sciences and Ziöra, and is a Director of Simbryo Technologies. A.C.-B. is an employee, shareholder, and company officer at Conceivable Life Sciences, and a shareholder in IVF 2.0. and Hope IVF Mexico. S.P. has received consultancy fees from Conceivable Life Sciences. S.K. is an employee, stock option holder, and company officer at Conceivable Life Sciences, has received consultancy fees from the WHO and Vitra Labs, and is Board Trustee of The King’s Fund. A.M. is a shareholder and company officer at Conceivable Life Sciences and holds shares in TMRW Life Sciences. D.S. is a shareholder and board member at Oova, a board member of AutoIVF, an advisory board member of Evvy, Conceivable Life Sciences, and The US Fertility venture fund, and a general partner at the Special Situations Life Sciences Fund and Life Sciences Innovation Fund I–II. G.D.A. is CEO of Advanced Reproductive Care Inc. (ARC Fertility), Chair of ICMART, and President of the World Endometriosis Research Foundation, and received consultancy fees from Pendant Health. Furthermore, G.S., A.C.-B., and A.M. declare inventorship on the US patents 12 178 475 B1 (automated sperm preparation) and 12 226 125 B2 (automated vitrification). A.C.-B. and A.M. declare inventorship on the US patents 12 349 940 B2 (automated oocyte denudation), 12 245 793 B2 (robotic handling systems for IVF laboratories), 12 180 441 B1 (laboratory workflow automation), 12 268 418 B2 (automated oocyte preparation), 12 310 625 B2 (automated ICSI), and 12 253 516 B2 (optical systems for IVF automation), as well as pending patent applications relating to automated dish preparation technology and automated vitrification systems.

**TRIAL REGISTRATION NUMBER:**

N/A.

WHAT DOES THIS MEAN FOR PATIENTS? The country a fertility patient lives in has a tremendous impact on their chances of being able to access IVF treatment. In some countries, more than 1 in 10 babies are now born through IVF; in others, fewer than one in a hundred. The difference is not explained by how many people struggle to conceive, but by how much treatment costs.

In this work, we looked at 22 countries covering most fertility treatments worldwide. We calculated the total cost of having a baby through IVF (including failed cycles) and expressed it as a share of the typical annual household income in each country. Our model predicts that halving the net amount patients have to pay would translate into roughly 2.7 times as many babies born through IVF.

Our work identified two concrete targets: bringing the underlying price of an IVF birth below 1 year of typical patient household income, through regulation, competition, or public provision; and bringing what patients actually pay below half a year’s income, through insurance, public funding, or tax relief. Countries achieving both, such as Israel, Japan, Spain, and South Korea, give their citizens demonstrably better access to IVF treatment.

## Introduction

Infertility affects approximately one in six people of reproductive age worldwide, with comparable prevalence across high-income and low-and-middle-income settings (WHO Infertility Prevalence Estimates 1990–2021, 2023). Infertility carries substantial psychological, social, and economic consequences for affected individuals and communities (Inhorn and Patrizio, 2015) ART, principally IVF, represents the most effective treatment for many forms of infertility, and global ART activity has expanded considerably over the past two decades. The most recent International Committee for Monitoring Assisted Reproductive Technologies world report documented approximately 3.3 million treatment cycles performed across 83 countries in 2017–2018 (Baker *et al.*, 2025). Yet this impressive aggregate figure conceals profound inequities in access.

ART utilization varies by more than an order of magnitude across countries of comparable economic development. Within Europe alone, treatment rates range from 142 to 3230 cycles per million inhabitants, and when expressed per million women of reproductive age, the spread widens further, from 753 (Malta) to 18 715 (Greece) (Smeenk *et al.*, 2025). Denmark, Belgium, and Israel consistently achieve the highest utilization globally, while countries including the USA, despite substantial clinical capacity, report rates well below estimated needs, resulting in an underserved population (Chambers *et al.*, 2009; Peipert *et al.*, 2023).

Already in 2007, Boivin *et al.* (2007) estimated that among 72.4 million infertile women worldwide, 56% sought medical help but only 22.4% received treatment, a disparity that Dyer *et al.* (2020) subsequently proposed quantifying through national ART utilization rates as a standardized indicator of access to infertility care. The International Federation of Fertility Societies (IFFS) surveillance programme has documented across successive editions that fewer than half of countries offering ART provide any public financial support, and only approximately one in five offers full reimbursement (International Federation of Fertility Societies’ Surveillance (IFFS), 2019, 2022). These data collectively describe a persistent global access gap of extraordinary scale.

Among the multiple factors contributing to this gap, including regulatory restrictions, cultural attitudes, clinical infrastructure, and physician availability; cost is consistently identified as the single most important barrier. Adamson (2009) identified economic factors and the absence of public funding as the principal determinants of international variation in ART access. Connolly *et al.* (2010), in a review for the ESHRE Reproduction and Society Task Force, concluded that only countries with funding arrangements that minimize out-of-pocket expenses approach estimated demand. In low-and-middle-income countries, the barrier is particularly stark: Njagi *et al.* (2023) found that a single IVF cycle frequently exceeds annual average income, constituting catastrophic health expenditure by standard definitions, while Chiware *et al.* (2021) reported ART services entirely absent or functionally inaccessible in many low- and middle-income countries due to prohibitive costs. The most recent IFFS consensus document reinforces the position plainly: affordability is the major barrier to access (Fauser *et al.*, 2024).

The foundational cross-national analysis in this area was performed by Chambers *et al.* (2009), who in 2009 compared IVF costs across six developed countries and found that a single cycle consumed ∼50% of annual disposable income in the USA but only 6% in Australia, a distinction driven largely by funding structures rather than raw clinic prices. This work was extended by the same group in 2014 by assessing national ART utilization against affordability defined as cycle cost expressed as a percentage of gross national disposable income per capita and demonstrated a statistically significant affordability gradient across countries (Chambers *et al.*, 2014). That analysis, now over a decade old, remains the closest precedent to a formal quantitative model linking affordability to utilization at the population level.

Several important limitations characterize the existing evidence base. First, prior analyses have relied on per-cycle cost as the measure of economic burden, yet patients typically require multiple cycles to achieve a live birth. Cumulative live birth rates are substantially higher than per-cycle rates. For example, Smith *et al.* (2015) demonstrated that first-cycle live birth rate in the UK was 29.5%, rising to 65.3% after six complete cycles, meaning the true cost to achieve a baby is two to three times higher than single-cycle cost, depending on patient age and prognosis. Keller and Chambers (2022) argued that cost-per-live-birth is the appropriate outcome metric for economic evaluation of fertility treatments, superseding both per-cycle cost and conventional Quality-Adjusted Life Year thresholds. Despite this, no cross-national affordability analysis has adopted cost-to-baby as the independent variable.

Second, existing studies have normalized treatment costs to disposable income per capita or gross domestic product, neither of which directly captures the economic capacity of the typical household. Median household income (HHI), being less distorted by income inequality than per-capita averages, more accurately reflects the affordability pressure experienced by the relevant decision-making unit.

Third, and perhaps most critically, no study has tested the functional form of the affordability–utilization relationship. In 2014, Chambers *et al.* (2014) employed ordinary least-squares regression implying a linear relationship; however, given that ART utilization spans more than an order of magnitude across countries, a power-law specification may provide a more parsimonious and empirically accurate description of the data.

Finally, the distinction between gross cost-to-baby (the full fee-for-service cost of achieving a live birth) and net out-of-pocket cost-to-baby (after insurance, subsidies, and tax benefits) has not been formally examined, despite the fact that two countries with identical gross costs may exhibit vastly different utilization rates if one subsidizes treatment heavily.

The present study aims to quantify the relationship between ART affordability defined as both gross and net cost-to-baby expressed as a percentage of median HHI and population-level ART utilization across 22 countries and regions representing more than 95% of global ART cycles. We hypothesize that this relationship follows a power-law function, implying constant elasticity of utilization with respect to affordability across the observed range. As a secondary aim, we seek to identify affordability thresholds associated with high and low utilization tiers, offering a data-driven framework for policymakers evaluating the likely impact of financial interventions on ART access within their populations.

## Materials and methods

### Study design

This is a cross-national observational study comparing country/region-level ART affordability metrics against population-level utilization outcomes.

### Data sources

ART cycle volumes and live birth outcomes were extracted from the International Committee for Monitoring Assisted Reproductive Technologies (ICMART) world reports (Chambers *et al.*, 2021; Baker *et al.*, 2025), the European Society of Human Reproduction and Embryology (ESHRE) (Smeenk *et al.*, 2025), the United States Center for Disease Control and Prevention National ART Surveillance System (CDC NASS) (CDC, 2022), the Canadian Assisted Reproductive Technologies Register (CARTR) (CARTR-Plus & BORN Ontario, 2025), UK’s Human Fertilisation and Embryology Authority (HFEA) (HFEA, 2025), the Latin American Network of Assisted Reproduction (REDLARA) (Zegers-Hochschild *et al.*, 2025), the Australian and New Zealand Assisted Reproduction Database (ANZARD) (Kotevski *et al*., 2023), and national registries where available (see Table 1), covering reporting years 2021–2023. The dataset encompasses 22 countries and regions representing more than 95% of estimated global ART cycle volume.

**Table 1.** Gross and net cost-to-baby (absolute and as % of median HHI), ART births as a percentage of total births, and ART births per 1000 women aged 15–49 years.

Country or territory	Median HHI	Annual ART cycles (n)	Livebirth/ART cycle	ART cycles/birth	Gross ART cost (USD)	Gross cost to baby	Gross cost to baby (%HHI)	Net cost to baby (%HHI)	% of ART births	ART cycles/1000 women	ART births/1000 women	Reference(s)	Data quality
Africa^¶^	$3140	7800	20.5%	4.9	$5349	$26 158	833%	825%	<0.1%	<0.1	0.02	Archary *et al.* (2024) Dyer *et al.* (2026)	Estim
AUS and NZ	$40 270	60 136	32.8%	3.0	$12 562	$38 267	95%	40%	5.6%	8.2	2.70	Kotevski *et al*. (2023)	High
Brazil	$9313	41 465	26.5%	3.8	$6375	$24 048	258%	236%	0.4%	0.7	0.20	Zegers-Hochschild *et al.* (2025)	High
Canada	$52 527	25 706	30.3%	3.3	$18 471	$60 961	116%	72%	2.2%	2.9	0.88	CARTR-Plus & BORN Ontario (2025)	High
China	$15 251	1 055 000	30.0%	3.3	$6509	$21 697	142%	103%	3.6%	3.3	0.99	Hu *et al.* (2020) Suzhou (2025)	Mod
East Europe	$14 482	190 805	36.1%	2.8	$6989	$19 336	134%	90%	1.7%	2.0	0.72	Smeenk *et al.* (2025)	Mod
Egypt	$4315	35 200	21.0%	4.8	$5360	$25 524	591%	574%	0.3%	1.2	0.26	Archary *et al.* (2024) Dyer *et al.* (2026)	Estim
India	$6358	225 000	24.0%	4.2	$4500	$18 758	295%	292%	0.2%	0.6	0.14	Ernst & Young LLP (2015)	Mod
Israel	$56 399	69 600	21.5%	4.6	$8017	$37 263	66%	13%	8.7%	32.6	7.00	Israel Ministry of Health (2024)	High
Japan	$27 556	275 296	26.0%	3.8	$5100	$19 619	71%	27%	9.3%	11.7	3.05	Katagiri *et al.* (2024)	Mod
Latin America^†^	$10 427	44 794	23.7%	4.2	$6383	$26 909	258%	222%	0.3%	0.6	0.15	Zegers-Hochschild *et al.* (2025)	Estim
Mexico	$10 427	20 659	29.1%	3.4	$6250	$21 498	206%	172%	0.4%	0.6	0.17	Zegers-Hochschild *et al.* (2025)	High
Middle East^‡^	$19 724	125 250	24.0%	4.2	$7649	$31 913	162%	104%	0.9%	2.3	0.56	Almaslami and Aljunid (2020)	Estim
SE Asia	$9229	115 000	24.9%	4.0	$6850	$27 468	298%	281%	0.3%	0.6	0.16	Abat *et al.* (2026)	Mod
South Africa	$8555	10 000	22.0%	4.5	$5850	$26 591	311%	294%	0.2%	0.6	0.13	Archary *et al.* (2024) Dyer *et al.* (2026)	Estim
South Korea	$30 222	129 286	21.6%	4.6	$7728	$35 832	119%	28%	11.8%	11.4	2.45	Lee *et al.* (2025)	Mod
Spain	$36 737	107 798	35.9%	2.8	$9862	$27 493	75%	52%	11.7%	10.5	3.76	Sociedad Española de Fertilidad (2024)	High
Taiwan, China	$31 852	84 205	19.7%	5.1	$8559	$43 408	136%	73%	11.9%	15.7	3.10	Taiwan Ministry of Health and Welfare (2024)	Mod
Tunisia	$10 262	7000	24.4%	4.1	$5584	$22 885	223%	213%	1.0%	2.3	0.55	Archary *et al.* (2024) Dyer *et al.* (2026)	Estim
UK	$42 588	74 801	27.5%	3.6	$15 985	$58 128	136%	99%	3.1%	5.0	1.37	HFEA (2025)	High
USA	$69 240	208 000	35.3%	2.8	$30 000	$84 986	123%	79%	2.5%	2.8	0.97	CDC (2022)	High
West Europe^§^	$37 978	349 651	33.6%	3.0	$10 535	$31 394	83%	38%	3.9%	5.4	1.82	Smeenk *et al.* (2025)	Mod

¶ Excluding Egypt, Tunisia, South Africa.

† Excluding Mexico and Brazil.

‡ Excluding Turkey, Israel, Egypt.

§ Excluding Spain, UK.

Data Quality: High = government, ART/Healthcare regulatory authorities and professional societies and 10 or more published 3rd party and clinic fee schedules sampled in country/region. Mod = Moderate. Grey literature and 3—5 published 3rd party and clinic fee schedules sampled in country/region. Estim = Estimate. Expert opinion based on relative comparison to similar countries with higher data quality. Source details are offered in Supplementary Tables S2 and S4. HHI, Household income; USD, US dollars.

Countries with sufficiently complete data from a dedicated national registry were analysed individually, whereas countries with merged, sparse or heterogeneous data were aggregated into regional groupings (Australia and New Zealand, Western Europe, Eastern Europe, the Middle East, South-East Asia, the rest of Latin America, and the rest of Africa). The full composition of each regional grouping is given in Supplementary Table S1.

ICMART estimation methods for total cycle volumes and live births, including extrapolation from participating to non-participating centres and confidence categorization based on cycle participation rates, were applied as described in the ICMART 2014 and ICMART 2017–2018 world reports (Chambers *et al.*, 2021; Baker *et al.*, 2025). Countries with fewer than 33% of cycles reported to ICMART and without an informed regional estimate were not included in utilization calculations.

Median after-tax disposable HHI was obtained from the Organisation for Economic Co-operation and Development (OECD) (OECD, 2026) and the World Bank (2021). In cases where only average HHI was available, the typical ratio of median to average (70%) from comparable countries was used to estimate the median HHI. Female population aged 15–49 years and total live births were sourced from the United Nations Population Division (United Nations, Department of Economic and Social Affairs, Population Division, 2024).

### Affordability metrics

Gross cost-to-baby was defined as the mean total fee-for-service cost of achieving one ART live birth, before any insurance reimbursement, government subsidy, or tax benefit. It was calculated as the product of mean cost per IVF cycle (from published clinic fee surveys and national fee schedules within each country/region), including embryo transfer, preimplantation genetic testing (PGT) (adjusted for usage rate in the given country/region), and medication costs, and the weighted mean number of cycles required to achieve a live birth, adjusted for regional maternal age distribution. Age adjustment was performed by applying age-specific cumulative live birth rates derived from registry data to the maternal age distribution observed in each country/region, yielding an age-weighted cycles-to-baby estimate.

Net out-of-pocket cost-to-baby was defined as the residual cost borne by the patient after accounting for private and government insurance coverage, direct subsidies, and applicable tax deductions or credits. For countries with published government reimbursement schedules including Japan (National Health Insurance), Australia (Medicare Benefits Schedule/Pharmaceutical Benefits Scheme), Israel (Health Basket), South Korea (National Health Insurance Service), and several European national health systems, subsidy values were derived directly from official sources. For countries where comprehensive published data were not available, net costs were estimated through structured triangulation of published literature against estimates obtained from grey literature, comparable countries, expert consultation, and interrogating language models GPT (version 4.0, OpenAI, San Francisco, CA, USA), Claude (version 4.5 Opus, Anthropic, San Francisco, CA, USA), and Gemini (version 1.5Pro, Google, Mountain View, CA, USA) to discover and synthetize information sources; these estimates are flagged in the results.

The underlying data and data sources are presented in Supplementary Tables S2, S3, S4, and S5.

Both gross and net cost-to-baby were expressed as a percentage of median HHI in the corresponding country/region. Treatment costs and HHI were expressed in nominal local-currency terms. Purchasing power parity adjustment was not applied because the affordability metric is constructed as a within-country ratio of local treatment cost to local HHI; it inherently reflects the economic reality faced by the household contemplating treatment. Such conversion would re-weight income against a standardized international consumption basket that does not correspond to the actual cost structure of fertility care.

### Utilization outcomes

ART utilization was assessed using two complementary measures.

- The relative measure was defined as the percentage of total national live births achieved via ART, capturing the technology’s contribution to the overall birth landscape.
- The absolute measure was defined as the number of ART-conceived live births per 1000 women of reproductive age (15–49 years), providing a population-standardized rate that is not confounded by differences in national birth rates.

Both measures were calculated from the same reporting period as the cost data.

### Statistical analysis

The relationship between affordability (cost-to-baby as percentage of median HHI) and utilization was assessed using power-law regression. This specification was selected on the basis that ART utilization spans more than an order of magnitude across the countries studied, suggesting a multiplicative rather than additive relationship: a constant proportional change in affordability produces a constant proportional change in utilization. In log-transformed space, this corresponds to a linear model of the form log(utilization) = α + β.log(affordability), where the slope coefficient β represents the elasticity of utilization with respect to affordability.

Log-linear models were fitted via ordinary least squares on transformed variables; the analyses were performed in Excel (version 16.0, Microsoft 365 MSO, Microsoft, Redmond, WA, USA). Pearson correlation coefficients were calculated from natural-log-transformed variables. Model fit was assessed by *R*^2^ values. Alternative functional forms were evaluated. Linear regression on untransformed variables yielded consistently lower *R*^2^ values across all four model specifications, confirming that the power-law (log–log) specification provides superior fit to the observed data.

A subgroup analysis was performed for women aged 38–49 years to evaluate whether the affordability–utilization relationship is amplified in an age group where cumulative live birth rates are lower and the number of cycles required to achieve a live birth and therefore cost-to-baby is substantially higher.

### Sensitivity analysis by Monte Carlo simulation

To quantify the impact of uncertainty in input estimates on the primary regression findings, a Monte Carlo probabilistic sensitivity analysis was performed in Microsoft Excel, following established practice in health economic evaluation (Claxton *et al.*, 2005; Briggs *et al.*, 2006; Cassettari *et al.*, 2016).

Four key input variables for each of the 22 countries and regions:

- mean gross IVF cycle cost (inclusive of one frozen embryo transfer, PGT weighted by national usage rate, and medications)
- mean number of cycles to live birth
- median HHI
- out-of-pocket cost as a percentage of gross cost-to-baby

were each assigned a PERT (Program Evaluation and Review Technique) probability distribution.

The PERT distribution, a four-parameter beta distribution defined by minimum, mode, and maximum values (Vose, 2008), was selected because it accommodates asymmetric uncertainty around a central estimate while producing a smooth, unimodal curve that concentrates probability near the most likely value, properties well suited to expert-derived cost and utilization inputs where full empirical distributions are unavailable. For each variable, the mode was set to the baseline point estimate.

Uncertainty bands were assigned to each country/region based on the quality and provenance of the underlying cost data, using a three-tier classification. High-quality sources (±5% uncertainty band) were defined as government publications, ART or healthcare regulatory authority reports, professional society data, and cost inputs corroborated by 10 or more independently published third-party or clinic fee schedules sampled within the country/region. Moderate-quality sources (±7.5% uncertainty band) comprised a combination of published and grey literature with three to five independently sampled fee schedules available for cross-validation. Estimated sources (±10% uncertainty band) relied on expert opinion informed by relative comparison with economically or structurally similar countries for which higher-quality data were available, supplemented by AI-assisted triangulation using large language models (OpenAI GPT, Anthropic Claude, Google Gemini) to discover and synthetize information sources. The tier assigned to each country/region is reported alongside the corresponding cost inputs in Table 1. A standard shape parameter of λ = 4 was applied throughout, yielding PERT shape parameters:


$$\alpha_{1}=1+\lambda \times (\mathrm{mode}-\min)/(\max-\min)\;\mathrm{and}\alpha_{2}=1+\lambda \times (\max-\mathrm{mode})/(\max-\min).$$

For each of the four input variables in each of the 22 countries and regions (88 distributions in total), 10 000 independent random values were drawn from the corresponding PERT distribution. Each draw was generated by sampling a uniform random number between 0 and 1 and transforming it through the inverse cumulative distribution function of the beta distribution with the country-specific shape parameters (α_1_, α_2_), then rescaling to the range defined by the minimum and maximum values (Vose, 2008).

In each simulation run, the two derived affordability metrics (gross cost-to-baby as a percentage of median HHI, and net out-of-pocket cost-to-baby as a percentage of median HHI) were recalculated for every country/region. The 5th and 95th percentile values (P5, P95) of each derived affordability metric were then extracted across the 10 000 runs for each country/region. Power-law regressions were fitted to the P5 and P95 affordability values against both utilization measures (percentage of births via ART, and ART births per 1000 women aged 15–49 years), generating 90% simulation envelopes around each of the four baseline regression models. These envelopes are presented graphically alongside the baseline regressions.

All analyses were performed in Microsoft Excel.

## Results

### The affordability landscape

Gross cost-to-baby varied more than 12-fold across the 22 countries and regions studied, ranging from 66% of median HHI in Israel to 833% in Africa (excluding Egypt, Tunisia, and South Africa). Net out-of-pocket cost-to-baby showed a similar spread, from 13% of median HHI in Israel to 825% in the same African grouping. The gap between gross and net costs was substantial in countries with comprehensive public funding or insurance coverage (e.g. Israel, Japan, Australia) and negligible in regions where patients bear the full fee-for-service cost. Table 1 presents gross and net cost-to-baby (absolute and as % of median HHI), ART births as a percentage of total births, and ART births per 1000 women aged 15–49 years for all 22 countries and regions.

### Affordability predicts utilization: power-law regression

Power-law regression revealed strong inverse relationships between affordability and ART utilization. The strongest associations were observed between gross cost-to-baby and ART births per 1000 women of reproductive age (*R*^2^ = 0.84) and between net out-of-pocket cost-to-baby and the same absolute utilization measure (*R*^2^ = 0.84). Both gross and net cost-to-baby also showed strong predictive value for the relative proportion of total births achieved via ART (*R*^2^ = 0.83 and *R*^2^ = 0.77, respectively). In all four models, the regression slope in log-transformed space was negative, confirming that higher cost burden is associated with lower utilization across the full range of observed values. The analysis is reported in Fig. 1.

**Figure 1.**
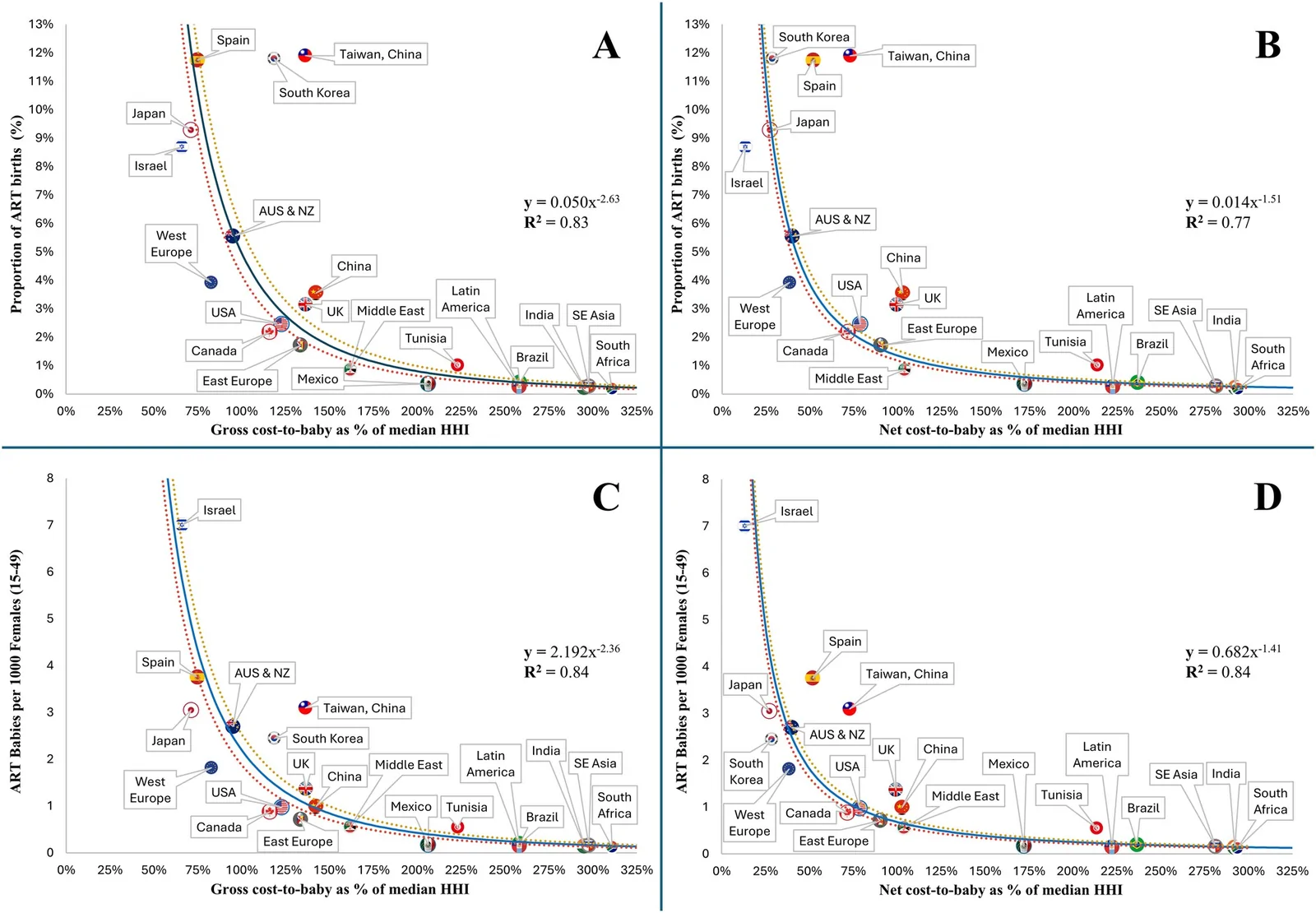
**Power-law regression analysis for all reproductive age women (15–49).** There was a strong inverse relationship between affordability and ART utilization for reproductive age women 15–49. This was evident when considering either gross or estimated net cost-to-baby. (**A**) Proportion of ART births versus Gross cost-to-baby as % of median HHI. (**B**) Proportion of ART births versus Net cost-to-baby as % of median HHI. (**C**) ART Babies per 1000 Females (15–49) versus Gross cost-to-baby as % of median HHI. (**D**) ART Babies per 1000 Females (15–49) versus Net cost-to-baby as % of median HHI. Blue continuous line = power-law regression line. Gold dotted line = 95th percentile confidence band. Red dotted line = 5th percentile confidence band. HHI, household income.

### Age subgroup analysis

The power-law relationship was preserved in women aged 38–49 years, with *R*^2^ values ranging from 0.76 to 0.85 across the four model specifications. The cost-to-baby burden in this age group was ∼1.5 to 2 times higher than in the overall study population, reflecting the lower cumulative live birth rates and greater number of cycles required to achieve a live birth in older women. The affordability–utilization gradient of the older sub-group was of comparable magnitude to the observed on the overall (all-age) population, indicating that the relationship is robust across the reproductive age spectrum. The analysis is reported in Fig. 2.

**Figure 2.**
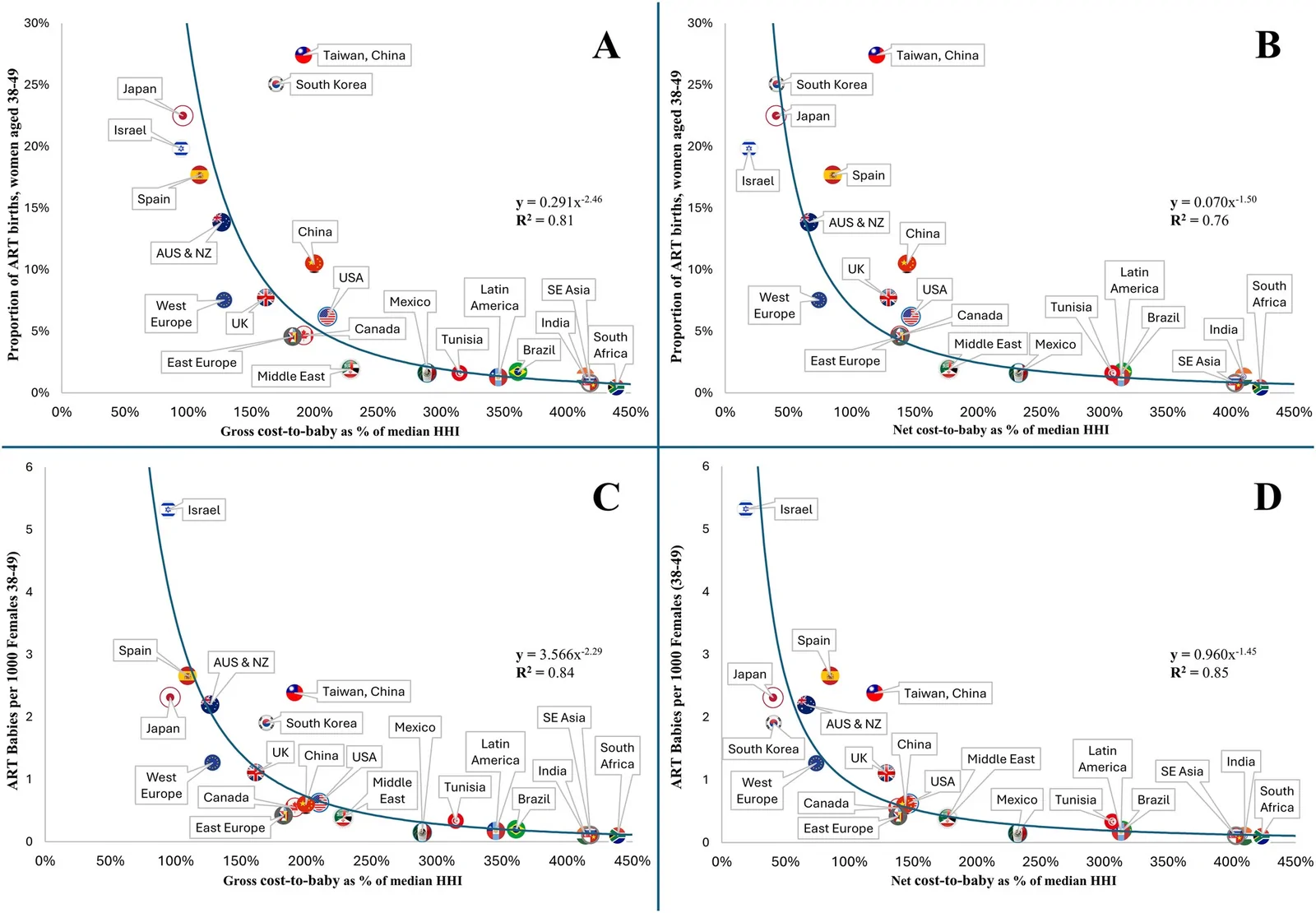
**Power-law regression analysis for women aged 38–49 years.** The inverse relationship between affordability and ART utilization is maintained. (**A**) Proportion of ART births versus Gross cost-to-baby as % of median HHI for women aged 38–49 years. (**B**) Proportion of ART births versus Net cost-to-baby as % of median HHI for women aged 38–49 years. (**C**) ART Babies per 1000 Females (38–49) versus Gross cost-to-baby as % of median HHI. (**D**) ART Babies per 1000 Females (38–49) versus Net cost-to-baby as % of median HHI. HHI, household income.

### Affordability tiers

Three utilization tiers emerged from the observed clustering of countries along the affordability–utilization gradient.

These tiers are descriptive groupings along a continuous affordability–utilization gradient rather than discrete categories with sharp boundaries: there is no distinct interface between high, moderate, and low utilization, and the bands therefore overlap, with the moderate-access zone spanning 80–120% of median HHI.

Regions where gross cost-to-baby fell below 100% of median HHI and net out-of-pocket cost-to-baby fell below 50% of median HHI achieved the highest ART utilization. Israel reported 8.7% of total births achieved via ART and 7.0 ART births per 1000 women of reproductive age; Japan 9.3% and 3.05; Spain 11.7% and 3.76; and South Korea 11.8% and 2.45.

Moderate-access regions, typified by gross costs of 80–120% and net costs of 40–75% of median HHI, included Australia/New Zealand (5.6% of births via ART; 2.70 per 1000 women) and Western Europe (3.9%; 1.82).

Taiwan, China was a notable outlier in this tier, achieving high-tier utilization (11.9%; 3.10) despite net costs in the moderate range, suggesting that additional policy or cultural factors may amplify utilization beyond what affordability alone would predict.

In low-access regions, where gross costs exceeded 120% of median HHI and net costs exceeded 75%, utilization was markedly lower. The USA reported 2.5% of all births via ART (0.97 ART births per 1000 women of reproductive age), and Latin American countries fell below 1% of all births via ART (∼0.15–0.20 ART births per 1000 women).

### Model-implied elasticities

The power-law specification yields constant-elasticity estimates of the affordability–utilization relationship. For net out-of-pocket cost-to-baby, the model implies that a 10% reduction in patient cost burden is associated with ∼16–17% higher utilization (measured as ART births per 1000 women of reproductive age), and that halving the cost burden is associated with a 2.67-fold increase in absolute utilization. The corresponding estimates for gross cost-to-baby are larger in magnitude, reflecting the steeper slope: a 10% reduction in gross cost is associated with an ∼28% increase in absolute utilization, and a 50% reduction predicts a 5.12-fold increase. The full elasticity estimates for all four model specifications are presented in Table 2.

**Table 2.** Elasticity estimates.

	10% cost reduction	25% cost reduction	50% cost reduction
Gross cost → % of births via ART	1.32×	2.13×	6.18×
Gross cost → ART births/1000 women	1.28×	1.97×	5.12×
Net cost → % of births via ART	1.17×	1.54×	2.85×
Net cost → ART births/1000 women	1.16×	1.50×	2.67×

These estimates were derived from the observed cross-sectional relationship and should not be interpreted as causal predictions of the effect of a specific policy intervention.

### Country-specific affordability targets

Using the power-law models, we calculated the reduction in gross and net cost-to-baby required for each country/region to reach 90th-percentile (best-in-class) utilization levels, defined as 11.5% of total births achieved via ART and 3.1 ART births per 1000 women of reproductive age. These country-specific estimates are presented in Table 3. As an example, shifting the USA from its current absolute utilization of 0.97 ART births per 1000 women to the 90th-percentile target of 3.1 would require reducing gross cost-to-baby by 30%, from 123% to 86% of median HHI. The corresponding model-implied net out-of-pocket cost-to-baby target for the USA is 34% of median HHI. Reaching this target would require action on both cost components: a reduction in gross cost-to-baby (a supply-side efficiency gain) would need to be accompanied by an expansion of insurance and subsidy coverage to bring net out-of-pocket cost-to-baby sufficiently down (a demand-side intervention).

**Table 3.** Country-specific affordability targets to reach 90th-percentile ART utilization (≥3.1 ART births per 1000 women of reproductive age).

Country or Territory	Required reduction in gross cost-to-baby (%)	Target net out of pocket as % of	
		Target gross cost-to-baby	Current gross cost-to-baby
Africa^¶^	90%	43%	4%
AUS and NZ	9%	43%	39%
Brazil	68%	43%	14%
Canada	40%	43%	26%
China	42%	43%	25%
East Europe	37%	43%	27%
Egypt	86%	43%	6%
India	72%	43%	12%
Israel	0%	53%	53%
Japan	0%	52%	52%
Latin America^†^	67%	43%	14%
Mexico	60%	43%	17%
Middle East^‡^	49%	43%	22%
SE Asia	72%	43%	12%
South Africa	74%	43%	11%
South Korea	32%	43%	29%
Spain	0%	46%	46%
Taiwan, China	40%	43%	26%
Tunisia	63%	43%	16%
UK	28%	43%	31%
USA	45%	43%	24%
West Europe^§^	10%	43%	39%

The table presents the required % reduction in gross cost-to-baby, and then the model-implied net out-of-pocket cost as a percentage of the reduced (target) gross cost. The last column expresses the same net target as a percentage of current gross cost-to-baby, capturing the combined effect of supply-side cost reduction and demand-side coverage expansion.

¶ Excluding Egypt, Tunisia, South Africa.

† Excluding Mexico and Brazil.

‡ Excluding Turkey, Israel, Egypt.

§ Excluding Spain, UK.

## Discussion

To our knowledge, this is the first study to quantify the relationship between ART affordability and population-level utilization using a cost-to-baby metric normalized to median HHI, and the first to identify a power-law functional form for this relationship. The consistency of the association across all four model specifications (*R*^2^ = 0.77–0.84), and across both gross and net affordability metrics, suggests that the cost of achieving a live birth, not the cost of a single cycle, is the economically relevant quantity governing treatment access at the population level. This metric also reflects the decision-making reality of patients, for whom the relevant economic question is not the cost of a single treatment cycle, but the total investment required to achieve a live birth.

Our analysis extends previous work (Chambers *et al.*, 2009, 2014) in three specific ways: adopting cost-to-baby rather than per-cycle cost as the affordability metric, normalizing to median HHI rather than per-capita disposable income, and testing a power-law functional form that yields constant-elasticity estimates directly comparable to the price-elasticity literature (Connolly *et al.*, 2009b). The result is a predictive model linking the funding landscape documented by the IFFS surveillance programme to the utilization outcomes tracked by ICMART and regional registries, through the mechanism of household-level affordability (Allan *et al.*, 2019; Horton *et al.*, 2022). The identified thresholds (gross cost-to-baby below 100% of median HHI, net below 50%) are observed characteristics of the highest-utilization countries.

That gross and net cost-to-baby independently predict utilization is among the most policy-relevant findings of this analysis, because the two metrics respond to fundamentally different interventions. Gross cost reflects the efficiency of the healthcare delivery system: clinic capacity, workforce models, technology adoption, and economies of scale. Net out-of-pocket cost reflects the policy environment: the extent to which insurance, subsidies, and tax incentives shield patients from the full fee-for-service burden.

While affordability is the overall binding constraint on ART access, neither lever alone appears sufficient to increase utilization. A country may subsidize treatment generously, but if gross costs are high, the fiscal burden limits coverage scope or eligibility; conversely, efficient low-cost clinics without public funding still place the full burden on household budgets. The countries achieving the highest utilization (Israel, Japan, Spain, South Korea) have acted on both levers simultaneously. In each high-utilization country, the combination of low gross costs and generous coverage brings net cost-to-baby below 50% of median HHI, crossing the threshold associated with high utilization in our model.

The French experience illustrates this asymmetry from the opposite direction. Despite near-complete public reimbursement, France does not achieve top-tier utilization and records among the highest rates of cross-border reproductive care in Europe, an outcome attributed to chronic underfunding of laboratory infrastructure and capacity constraints that effectively inflate the gross cost of treatment delivery (Chaillot *et al.*, 2026). In the framework of the present analysis, France is a case where the demand-side lever has been maximally deployed while the supply-side lever remains largely unaddressed. Indeed, expanding access to fertility treatment is also a capacity problem. Subsidies and insurance mandates cannot help patients if the industry lacks the infrastructure to treat them (Sable, 2026).

Taiwan presents an instructive outlier: despite net costs in the moderate range (40–75% of median HHI), it achieved utilization rates comparable to the highest-access tier (11.9% of births via ART), suggesting that cultural acceptance, regulatory accessibility, and the momentum of the 2021 subsidy expansion can amplify utilization beyond what affordability alone would predict. This does not undermine the model but illustrates that the power-law relationship captures dominant variance while leaving room for country-specific factors to shift.

The affordability targets presented here present a rationale for increased public expenditure. Whether such investment is fiscally defensible is addressed by a growing body of accounting analyses across different fiscal regimes, which was pioneered by Connolly (2026) and which project the lifetime fiscal profile of an ART-conceived individual. Across 12 countries studied, 11 show consistently positive returns: from 2.7× in Australia (Connolly *et al.*, 2025), to 5.7× in Taiwan, China (Chen *et al.*, 2025), and ∼16× in Spain (Matorras *et al.*, 2015), with comparable findings in middle-income settings like South Africa, 5.6 × (Connolly *et al.*, 2021), and earlier estimates for the USA of ∼7 × (Connolly *et al.*, 2008), UK 8.5 × (Connolly *et al.*, 2009a), Denmark of ∼14 × (Connolly *et al.*, 2011), and Sweden at 1.2 × (Svensson *et al.*, 2008). The sole exception, the Netherlands, reflects a welfare system that produces negative fiscal balances for all citizens, not specifically ART-conceived individuals (Moolenaar *et al.*, 2014). The consistency of positive returns across diverse settings supports the conclusion that ART represents a high-return public health investment in the context of demographic contraction (Connolly *et al.*, 2023; Fauser *et al.*, 2024). This urgency is underscored by the observation that more than half of the world’s nations already exhibit sub-replacement total fertility rates, with projections suggesting ∼97% will do so by the end of the century (Bhattacharjee *et al.*, 2024; Aitken, 2026). In countries where ART-conceived births already account for 7–10% of all newborns, ART has become de facto demographic infrastructure, yet the affordability barriers identified in the present analysis mean that this capacity remains inaccessible to most.

### Strengths and limitations

Readers should note that this is a cross-national, cross-sectional observational study based on aggregated, country-level data. The observed associations cannot support individual-level causal inference: relationships that hold across populations need not hold within them (ecological fallacy), and unmeasured country-level factors correlated with both affordability and utilization may contribute to the observed gradient. Accordingly, the elasticity estimates presented in Table 2 describe the observed cross-sectional relationship and should not be interpreted as forecasts of the effect of any specific policy intervention.

Registry data are heterogeneous across countries in reporting standards, cycle definitions, and completeness, as documented by ICMART and ESHRE (Baker *et al.*, 2025; Smeenk *et al.*, 2025). Unmeasured confounders include cultural and religious attitudes, regulatory restrictions on access, clinic capacity constraints, and cross-border reproductive care, which may inflate utilization estimates in destination countries and deflate them in origin countries. The analysis likewise did not capture the broader societal context of family formation, including family-friendly employment practices, parental leave, childcare provision, and the healthcare and educational support available to families which shape household reproductive decision-making independently of the direct cost of treatment. A reinforcement loop, where high utilization drives down costs through economies of scale, represents a potential source of reverse causation that a cross-sectional design cannot resolve. Moreover, the macro-level framework cannot resolve within-country racial or ethnic inequities in ART access, and this represents an important direction for future research.

Transparency regarding the provenance and quality of input data is also essential to interpreting these findings. ART utilization data derive from established international and national registries with documented quality assurance processes, and economic and demographic data from authoritative agencies (OECD, World Bank, UN). Gross cost inputs were drawn from published surveys and national fee schedules for the majority of countries. However, for a subset of countries and regional aggregates where comprehensive published data were not available from a single authoritative source, cost estimates—particularly net out-of-pocket costs—were derived through structured triangulation of expert consultation, grey literature, and AI-assisted synthesis using large language models (OpenAI GPT-4o, Anthropic Claude, Google Gemini Pro). These estimates are identified in the results section and supplementary tables and should be interpreted with appropriate caution.

This heterogeneity in input quality is the principal reason we performed Monte Carlo probabilistic sensitivity analysis with uncertainty bands explicitly calibrated to data provenance: ±5% for countries with well-documented published sources, ±7.5% for those with moderate-quality evidence, and ±10% for countries relying on estimated inputs. The assignment of countries to these tiers involved a semi-qualitative interpretation, and the specific band widths are not empirically derived; the three-tier scheme may understate uncertainty for the weakest sources and overstate it for the strongest, but it represents a structured approach to differential uncertainty that avoids embedding additional unverifiable assumptions. The critical finding is that the power-law relationship and the associated *R*^2^ values are not sensitive to plausible variation in any single country’s input estimates: the 90% simulation envelopes remain narrow across all four model specifications, indicating that the affordability–utilization gradient is a robust structural feature of the data and is not dependent on any individual point estimate.

Two further caveats apply. The simulation evaluates uncertainty in the independent variables (affordability metrics) while holding the dependent variables (utilization outcomes) fixed; utilization data also carry measurement uncertainty, and the reported envelopes should therefore be interpreted as sensitivity bands on the affordability estimates rather than full probabilistic confidence intervals on the fitted relationship. The practical implication is that even if specific country-level cost estimates carry meaningful uncertainty the overall relationship and the policy-relevant thresholds derived from it are stable.

Nevertheless, we call for the development of a standardized international ART affordability monitoring framework, analogous to the ICMART cycle reporting infrastructure, to replace estimated inputs with verified data over time.

## Conclusions

Affordability measured as the cost of achieving an ART live birth relative to median HHI follows a power-law relationship with population-level utilization. The metric proposed here offers a standardized framework for benchmarking fertility care access globally. Two complementary economic levers can increase utilization: reducing gross cost-to-baby below 100% of median HHI through provider-side efficiencies and reducing net out-of-pocket cost-to-baby below 50% of median HHI through coverage expansion, subsidies, or tax incentives. Countries that have acted on both levers simultaneously achieve the highest utilization globally. The power-law nature of the relationship implies that the marginal return on affordability improvements is greatest where costs are currently highest relative to income.

## Supplementary Material

hoag084_Supplementary_Data

## Data Availability

All data used in this study were derived from publicly available secondary sources. The complete country-level dataset underpinning the analyses, including gross and net cost-to-baby estimates, median HHI values, ART utilization measures, data source classifications, and uncertainty band assignments for all 22 countries and regions, are available as supplementary tables. The Monte Carlo simulation parameters (PERT distribution specifications for each country/region and variable) will be made available upon reasonable request to the corresponding author. No individual participant data were collected or used in this study. No additional documents (study protocol, statistical analysis plan, informed consent form) are applicable, as this is a secondary analysis of aggregate publicly available data. Data will be available from the date of publication for 36 months. Access will be granted to researchers who provide a methodologically sound proposal, without requirement for a signed data access agreement, for any legitimate research purpose.

## References

[hoag084-B1] Abat MAG , Armstrong H, Chen M-J, Fatemi H, Hung HS, Nakagawa K, Pannu NS, Petrova A, Pistollato M, Pruksananonda K et al Improving access to fertility treatment in Asia Pacific: consensus from an expert forum. Front Reprod Health 2026;8:1738726. 10.3389/frph.2026.1738726 PMC13047160 41938997

[hoag084-B2] Adamson GD. Global cultural and socioeconomic factors that influence access to assisted reproductive technologies. Womens Health (Lond) 2009;5:351–358. 10.2217/whe.09.28 19586427

[hoag084-B3] Aitken RJ. Declining global fertility and our future dependence on ART. Reprod Biomed Online 2026;53:105697. 10.1016/j.rbmo.2026.105697 42048936

[hoag084-B5] Almaslami F , Aljunid SM. Cost-effectiveness of assisted reproductive technologies in Saudi Arabia: comparing in vitro fertilization with intrauterine insemination. SAGE Open Med 2020;8:2050312120931988. 10.1177/2050312120931988 PMC7294478 32587695

[hoag084-B7] Archary P , Potgieter L, Honwana F, Elgindy E, Adageba RK, Coulibaly FA, Iketubosin F, Serour G, Dyer S, Network A; African Network and Registry for Assisted Reproductive Technology. Assisted reproductive technologies in Africa: the African network and registry for ART, 2020. Reprod Biomed Online 2024;49:104353. 10.1016/j.rbmo.2024.104353 39305801

[hoag084-B8] Baker VL , Dyer S, Chambers GM, Keller E, Banker M, Mouzon JD, Elgindy E, Bai FM, Ishihara O, Jwa SC et al International Committee for Monitoring Assisted Reproductive Technologies (ICMART): world report for cycles conducted in 2017–2018. Hum Reprod 2025;40:1110–1126. 10.1093/humrep/deaf049 40239109

[hoag084-B9] Bhattacharjee NV , Schumacher AE, Aali A, Abate YH, Abbasgholizadeh R, Abbasian M, Abbasi-Kangevari M, Abbastabar H, Abd ElHafeez S, Abd-Elsalam S et al Global fertility in 204 countries and territories, 1950–2021, with forecasts to 2100: a comprehensive demographic analysis for the Global Burden of Disease Study 2021. Lancet 2024;403:2057–2099. 10.1016/S0140-6736(24)00550-6 PMC11122687 38521087

[hoag084-B10] Boivin J , Bunting L, Collins JA, Nygren KG. International estimates of infertility prevalence and treatment-seeking: potential need and demand for infertility medical care. Hum Reprod 2007;22:1506–1512. 10.1093/humrep/dem046 17376819

[hoag084-B11] Briggs A , Sculpher M, Claxton K. Decision Modelling for Health Economic Evaluation, 1st edn. Oxford, UK: Oxford University Press, 2006.

[hoag084-B12] *[Dataset]* CARTR-Plus & BORN Ontario. *Canadian Assisted Reproductive Technologies Register (CARTR) Plus Annual Report*. Canadian Fertility and Andrology Society, Dorval, QC, Canada. 2025. https://cfas.ca/cgi/page.cgi/cartr-reports.html (January 2026, date last accessed).

[hoag084-B13] Cassettari L , Mosca M, Mosca R, Rolando F, Costa M, Pisaturo V. IVF cycle cost estimation using activity based costing and Monte Carlo simulation. Health Care Manag Sci 2016;19:20–30. 10.1007/s10729-014-9282-2 24752546

[hoag084-B14] *[Dataset]* CDC. *Assisted Reproductive Technology (ART) Success Rates 2022*. Centers for Disease Control and Prevention, Atlanta, GA, USA. 2022. https://art.cdc.gov/artclinics (January 2026, date last accessed).

[hoag084-B15] Chaillot M , Pollet-Villard X, Sanguinet P, Lefleuter N, Agopiantz M, Bachelot G, Brouillet S, Fréour T. Universal coverage of IVF: benefits, unintended barriers and lessons from the French model in an international perspective. Reprod Biomed Online 2026;52:105542. 10.1016/j.rbmo.2026.105542 41932286

[hoag084-B16] Chambers GM , Dyer S, Zegers-Hochschild F, Mouzon JD, Ishihara O, Banker M, Mansour R, Kupka MS, Adamson GD. International Committee for Monitoring Assisted Reproductive Technologies world report: assisted reproductive technology, 2014. Hum Reprod 2021;36:2921–2934. 10.1093/humrep/deab198 34601605

[hoag084-B17] Chambers GM , Hoang VP, Sullivan EA, Chapman MG, Ishihara O, Zegers-Hochschild F, Nygren KG, Adamson GD. The impact of consumer affordability on access to assisted reproductive technologies and embryo transfer practices: an international analysis. Fertil Steril 2014;101:191–198.e4. 10.1016/j.fertnstert.2013.09.005 24156958

[hoag084-B18] Chambers GM , Sullivan EA, Ishihara O, Chapman MG, Adamson GD. The economic impact of assisted reproductive technology: a review of selected developed countries. Fertil Steril 2009;91:2281–2294. 10.1016/j.fertnstert.2009.04.029 19481642

[hoag084-B19] Chen M-J , Kotsopoulos N, Ming-Fang Yen A, Lin K-T, Connolly MP. Estimating the public economic gains in Taiwan from in vitro fertilization (IVF) subsidy changes implemented in 2021. Hum Reprod 2025;40:328–334. 10.1093/humrep/deae271 PMC11788206 39673722

[hoag084-B20] Chiware TM , Vermeulen N, Blondeel K, Farquharson R, Kiarie J, Lundin K, Matsaseng TC, Ombelet W, Toskin I. IVF and other ART in low- and middle-income countries: a systematic landscape analysis. Hum Reprod Update 2021;27:213–228. 10.1093/humupd/dmaa047 PMC7903111 33238297

[hoag084-B21] Claxton K , Sculpher M, McCabe C, Briggs A, Akehurst R, Buxton M, Brazier J, O’Hagan T. Probabilistic sensitivity analysis for NICE technology assessment: not an optional extra. Health Econ 2005;14:339–347. 10.1002/hec.985 15736142

[hoag084-B22] Connolly MP. The economics of hope: valuing the long-term fiscal returns from assisted reproduction. Reprod Biomed Online 2026;53:105738. 10.1016/j.rbmo.2026.105738 42202705

[hoag084-B23] Connolly M , Gallo F, Hoorens S, Ledger W. Assessing long-run economic benefits attributed to an IVF-conceived singleton based on projected lifetime net tax contributions in the UK. Hum Reprod 2009a;24:626–632. 10.1093/humrep/den435 19056775

[hoag084-B24] Connolly MP , Griesinger G, Ledger W, Postma MJ. The impact of introducing patient co-payments in Germany on the use of IVF and ICSI: a price-elasticity of demand assessment. Hum Reprod 2009b;24:2796–2800. 10.1093/humrep/dep260 19625316

[hoag084-B25] Connolly MP , Hoorens S, Chambers GM; ESHRE Reproduction and Society Task Force. The costs and consequences of assisted reproductive technology: an economic perspective. Hum Reprod Update 2010;16:603–613. 10.1093/humupd/dmq013 20530804

[hoag084-B26] Connolly MP , Kotsopoulos N, Eisenberg ML. The intergenerational economics of infertility, childrearing, and assisted reproduction. Fertil Steril 2023;119:184–185. 10.1016/j.fertnstert.2022.12.011 36502934

[hoag084-B27] Connolly M , Kotsopoulos N, Li J, Chambers G. Estimating the fiscal value of children conceived from assisted reproduction technology in Australia applying a public economic perspective. J Health Econ Outcomes Res 2025;12:148–154. 10.36469/001c.133796 PMC12009558 40255453

[hoag084-B28] Connolly MP , Panda S, Mburu G, Matsaseng T, Kiarie J. Estimating the government public economic benefits attributed to investing in assisted reproductive technology: a South African case study. Reprod Biomed Soc Online 2021;12:14–21. 10.1016/j.rbms.2020.08.001 PMC7533353 33033757

[hoag084-B29] Connolly MP , Pollard MS, Hoorens S, Kaplan BR, Oskowitz SP, Silber SJ. Long-term economic benefits attributed to IVF-conceived children: a lifetime tax calculation. Am J Manag Care 2008;14:598–604. 18778175

[hoag084-B30] Connolly MP , Postma MJ, Crespi S, Andersen AN, Ziebe S. The long-term fiscal impact of funding cuts to Danish public fertility clinics. Reprod Biomed Online 2011;23:830–837. 10.1016/j.rbmo.2011.09.011 22033399

[hoag084-B31] Dyer S , Chambers GM, Adamson GD, Banker M, De Mouzon J, Ishihara O, Kupka M, Mansour R, Zegers-Hochschild F. ART utilization: an indicator of access to infertility care. Reprod Biomed Online 2020;41:6–9. 10.1016/j.rbmo.2020.03.007 32448672

[hoag084-B32] Dyer S , Potgieter L, Honwana F, Elgindy E, Adageba RK, Khrouf M, Kolani JC, Iketubosin F, Le Roux P, Archary P; African Network and Registry for Assisted Reproductive Technology. Assisted reproductive technologies in Africa: the African Network and Registry for ART, 2021 and 2022. Reprod Biomed Online 2026;52:105230. 10.1016/j.rbmo.2025.105230 41483782

[hoag084-B33] Ernst & Young LLP. *Call for Action: Expanding IVF Treatment in India*. Mumbai, India. 2015. https://www.scribd.com/document/415017208/EY-Call-for-Action-Expanding-Ivf-Treatment-in-India (January 2026, date last accessed).

[hoag084-B34] Fauser BC , Adamson GD, Boivin J, Chambers GM, Geyter C, de Dyer S, Inhorn MC, Schmidt L, Serour GI, Tarlatzis B et al; Contributors and Members of the IFFS Demographics and Access to Care Review Board. Declining global fertility rates and the implications for family planning and family building: an IFFS consensus document based on a narrative review of the literature. Hum Reprod Update 2024;30:153–173. 10.1093/humupd/dmad028 PMC10905510 38197291

[hoag084-B35] *[Dataset]* HFEA. Fertility Treatment 2023: Trends and Figures. Human Fertilisation and Embryology Authority, London, UK. 2025. https://www.hfea.gov.uk/about-us/publications/research-and-data/fertility-treatment-2023-trends-and-figures/ (January 2026, date last accessed).

[hoag084-B37] Hu L , Bu Z, Huang G, Sun H, Deng C, Sun Y. Assisted reproductive technology in China: results generated from data reporting system by CSRM from 2013 to 2016. Front Endocrinol (Lausanne) 2020;11:458. 10.3389/fendo.2020.00458 PMC7527788 33042000

[hoag084-B38] Inhorn MC , Patrizio P. Infertility around the globe: new thinking on gender, reproductive technologies and global movements in the 21st century. Hum Reprod Update 2015;21:411–426. 10.1093/humupd/dmv016 25801630

[hoag084-B4] International Federation of Fertility Societies’ Surveillance (IFFS). International federation of fertility societies’ surveillance (IFFS) 2019: global trends in reproductive policy and practice, 8th edition. Glob Reprod Health 2019;4:1–138.

[hoag084-B36] International Federation of Fertility Societies’ Surveillance (IFFS).International Federation of Fertility Societies’ Surveillance (IFFS) 2022: global trends in reproductive policy and practice, 9th edition. Glob Reprod Health 2022;7:e58.

[hoag084-B39] *[Dataset]* Israel Ministry of Health. In vitro fertilization (IVF) treatments 1990-2022. Israel Ministry of Health, West Jerusalem, Jerusalem District, Israel. 2024. https://www.gov.il/BlobFolder/reports/ivf-reports/he/files_publications_units_info_IVF1990-2022.pdf (January 2026, date last accessed).

[hoag084-B40] Katagiri Y , Jwa SC, Kuwahara A, Iwasa T, Ono M, Kato K, Kishi H, Kuwabara Y, Taniguchi F, Harada M et al Assisted reproductive technology in Japan: a summary report for 2022 by the Ethics Committee of the Japan Society of Obstetrics and Gynecology. Reprod Med Biol 2024;23:e12620. 10.1002/rmb2.12620 PMC11646354 39677328

[hoag084-B41] Keller E , Chambers GM. Valuing infertility treatment: why QALYs are inadequate, and an alternative approach to cost-effectiveness thresholds. Front Med Technol 2022;4:1053719. 10.3389/fmedt.2022.1053719 PMC9822722 36619344

[hoag084-B6] *[Dataset]* Kotevski DP, Newman JE, Chaitarvornkit A, Paul RC, Chambers GM. Assisted reproductive technology in Australia and New Zealand 2023. Sydney: National Perinatal Epidemiology and Statistics Unit, the University of New South Wales, Sydney 2025. https://www.unsw.edu.au/content/dam/pdfs/medicine-health/npesu/research-reports/2025-09-anzard/2025-09-Assisted-Reproductive-Technology-in-Australia-and-New-Zealand-2023.pdf (January 2026, date last accessed).

[hoag084-B42] Lee D , Seong ME, Chang HJ, Choi YS, Lim HJ, Choi Y, Hwang K, Jo JS, Lee JR. Assisted reproductive technology trends in Korea: annual report for 2022. Clin Exp Reprod Med 2025;52:299–309. 10.5653/cerm.2025.08872 PMC12682406 41354030

[hoag084-B43] Matorras R , Villoro R, González-Domínguez A, Pérez-Camarero S, Hidalgo-Vega A, Polanco C. Long-term fiscal implications of funding assisted reproduction: a generational accounting model for Spain. Reprod Biomed Soc Online 2015;1:113–122. 10.1016/j.rbms.2016.04.001 PMC6001347 29911192

[hoag084-B44] Moolenaar LM , Connolly M, Huisman B, Postma MJ, Hompes PG, F van der V, Mol B. Costs and benefits of individuals conceived after IVF: a net tax evaluation in The Netherlands. Reprod Biomed Online 2014;28:239–245. 10.1016/j.rbmo.2013.10.002 24365025

[hoag084-B45] Njagi P , Groot W, Arsenijevic J, Dyer S, Mburu G, Kiarie J. Financial costs of assisted reproductive technology for patients in low-and middle-income countries: a systematic review. Hum Reprod Open 2023;2023:hoad007. 10.1093/hropen/hoad007 PMC10029849 36959890

[hoag084-B46] *[Dataset]* OECD. *OECD Data Explorer Income Distribution Database*. Organisation for Economic Co-operation and Development, Paris, France. 2026. https://data-explorer.oecd.org/vis?tm=income&pg=0&snb=462&df[ds]=dsDisseminateFinalDMZ&df[id]=DSD_WISE_IDD%40DF_IDD&df[ag]=OECD.WISE.INE (January 2026, date last accessed).

[hoag084-B47] Peipert BJ , Adashi EY, Penzias A, Jain T. Global in vitro fertilization utilization: how does the United States compare? F S Rep 2023;4:326–327. 10.1016/j.xfre.2023.06.005 PMC10504530 37719106

[hoag084-B48] Sable D. A plan for universal access to fertility care. Reprod Biomed Online 2026;53:105780. 10.1016/j.rbmo.2026.105780 42314454

[hoag084-B49] Smeenk J , Wyns C, De Geyter CH, Kupka MS, Bergh C, Cuevas Saiz I, De Neubourg D, Rezabek K, Tandler-Schneider A, Rugescu I et al ART in Europe, 2020: results generated from European registries by ESHRE†. *Hum Reprod* 2025;40:2038–2055. 10.1093/humrep/deaf179 40985526

[hoag084-B50] Smith AD , Tilling K, Nelson SM, Lawlor DA. Live-birth rate associated with repeat in vitro fertilization treatment cycles. JAMA 2015;314:2654–2662. 10.1001/jama.2015.17296 PMC4934614 26717030

[hoag084-B51] *[Dataset]* Sociedad Española de Fertilidad. *Informe estadístico de técnicas de reproducción asistida 2022*. Sociedad Española de Fertilidad, Madrid, Spain. 2024. https://www.registrosef.com/public/docs/sef2022_IAFIV.pdf (January 2026, date last accessed).

[hoag084-B52] Suzhou Basecare Corporation Ltd. The Rise of Advanced Maternal Age IVF: BaseCare Medical’-B(2170.HK) AI “Undisturbed Culture” Empowers Intelligent Assisted Reproduction. *PR Newswire*, 2025. https://www.prnewswire.com/news-releases/the-rise-of-advanced-maternal-age-ivf-basecare-medical-b2170hk-ai-undisturbed-culture-empowers-intelligent-assisted-reproduction-302620107.html (January 2026, date last accessed).

[hoag084-B53] Svensson A , Connolly M, Gallo F, Hägglund L. Long-term fiscal implications of subsidizing in-vitro fertilization in Sweden: a lifetime tax perspective. Scand J Public Health 2008;36:841–849. 10.1177/1403494808095086 19004901

[hoag084-B54] *[Dataset]* Taiwan Ministry of Health and Welfare. *The Assisted Reproductive Technology Summary 2022 National Report of Taiwan*. Taipei City, Taiwan: Taiwan Ministry of Health and Welfare. 2024. https://www.hpa.gov.tw/Pages/ashx/GetFile.ashx?lang=e&type=1&sid=3d2f51aee41b4648be6c3cba3318e6b4 (January 2026, date last accessed).

[hoag084-B55] *[Dataset]* United Nations, Department of Economic and Social Affairs, Population Division. *World Population Prospects 2024.* United Nations, Department of Economic and Social Affairs, Population Division, New York City, NY, USA. 2024. https://population.un.org/wpp/ (January 2026, date last accessed).

[hoag084-B56] Vose D. Risk Analysis: A Quantitative Guide, 3rd edn. Chichester, UK: John Wiley & Sons, 2008.

[hoag084-B57] WHO Infertility Prevalence Estimates 1990–2021. World Health Organization, Geneva, Switzerland. 2023. https://www.who.int/publications/i/item/978920068315 (January 2026, date last accessed).

[hoag084-B58] *[Dataset]* World Bank. *Adjusted Net National Income Per Capita (Current US$) 1970–2021*. World Bank, Washington, DC, USA. 2021. https://data.worldbank.org/indicator/NY.ADJ.NNTY.PC.CD (January 2026, date last accessed).

[hoag084-B59] Zegers-Hochschild F , Crosby JA, Musri C, Petermann-Rocha F, Martinez G, Nakagawa H, Morente C, Roque A, Palma-Govea A; Latin American Network of Assisted Reproduction. Assisted reproductive technologies in Latin America: the Latin American Registry, 2021. Reprod Biomed Online 2025;50:104413. 10.1016/j.rbmo.2024.104413 39537522

